# Assessment of orthotic needs in Iranian veterans with ankle and foot disorders

**DOI:** 10.1186/s40779-018-0159-4

**Published:** 2018-04-20

**Authors:** Kamiar Ghoseiri, Mostafa Allami, Mohammad Reza Soroush

**Affiliations:** 1 Department of Orthotics and Prosthetics, School of Rehabilitation Sciences, Hamadan University of Medical Sciences, Shahid Fahmideh Ave, Hamadan, Iran; 2 Janbazan Medical and Engineering Research Center (JMERC), Farrokh Ave, Velenjak, Tehran, Iran

**Keywords:** Needs assessment, Orthotic devices, Veterans, Ankle, Foot, War-related injuries

## Abstract

**Background:**

War-related traumas can lead to orthopedic and neurological disorders in victims. However, the scope of such disorders may expand months or even years after the trauma. Orthotic treatment as a rehabilitation process aims to enable people with disabilities to reach and maintain their optimal physical, sensory, intellectual, psychological, and social functional levels. This study aimed to investigate the rate of using orthoses among Iranian veterans with neuromuscular and skeletal disorders of the ankle and foot. Furthermore, the priorities of orthotic treatment in those veterans were explored.

**Methods:**

This qualitative study was a national health needs assessment conducted in 11 provinces of Iran during 2011–2016. A stakeholder research group was established to survey the veterans in respect to their demographic variables, activities of daily living, current health conditions, and rate of using orthoses.

**Results:**

Overall, 907 of the 1124 veteran participants completed the survey (response rate: 80.7%). Most of the veterans were men (97.7%), and their age and disability rate were 52.07 ± 8.13 years and 31.92% ± 14.93%, respectively. Nearly 42% of the veterans had experience in using orthoses on a daily and weekly basis. As physical ambulation was the main problematic activity in veterans, most of them were using medical shoes and foot orthoses. Nearly 37% of veterans were in need of some type of lower limb orthoses on the contralateral side to compensate for their hip inequality. In sequential order, the most in need orthoses for veterans were foot orthoses (*n* = 538), medical shoes (*n* = 447), lower limb orthoses on the contralateral side (*n* = 320), spinal orthoses (*n* = 273), and upper limb orthoses (*n* = 86).

**Conclusions:**

In spite of the high demands for orthoses among Iranian veterans with ankle and foot disorders, the use of orthoses is insufficient. Hence, there is a discrepancy between the current rate of orthoses use and its ideal situation, and more resources should be provided for service providers to be able to serve veterans. Moreover, veterans should be educated regarding orthoses, their use, and their impacts on the user’s health status. The findings of a needs assessment of orthoses can be used in strategic planning and decision making to improve health care services for Iranian veterans.

## Background

War-related traumas can lead to orthopedic and neurological disorders in victims. However, the scope of such disorders may expand months or even years after the trauma [1]. The quality of life, independence in daily activities, and overall satisfaction of life may be decreased in military and civilian veterans due to war-related traumas [2]. Furthermore, the aforementioned sequelae may cause psychological disorders in victims. Musculoskeletal injuries account nearly for 70% of all war-related injuries. The incidence of musculoskeletal injuries has a ratio of 3:2 of lower limbs to upper limbs [3, 4]. When these injuries are isolated, they mainly provide some type of disability or morbidity instead of mortality for victims [4].

The Iraq-Iran war (1980–1988) was one of the longest wars of the twentieth century and spanned the western and southwestern borders of Iran. It caused nearly 219,000 deaths, hundreds of thousands injured people, displaced millions, and caused billions of dollars in destruction in Iran [1]. War victims in Iran are under the support of the Veterans and Martyrs Affairs Foundation (VMAF). Based on the VMAF database, there are 548,499 veterans who live in Iran and suffer from chemical and traumatic war-related injuries. Of those, 700 have two-eye blindness, 153 have bilateral upper limb amputation, 2723 have unilateral upper limb amputation, 800 have bilateral lower limb amputation, and 11,776 have unilateral lower limb amputation [5].

The health needs and priorities of people with some kind of disability can be assessed systematically using multi-stakeholder focus-group workshops and remote monitoring technologies [6]. In this process, epidemiologic, qualitative, and comparative methods may be used to describe people’s health status, the delivery of services, and the accessibility of services in different regions of a territory. Health needs assessment is an evidence-based practice that starts with a deep clinical understanding of the current health status of people and their health care demands. Moreover, by providing an estimate of resources and allocated times, this assessment can lead to the proper planning of strategies and programs to improve health care services [7].

Orthotic treatment as a rehabilitation process aims to enable people with disabilities to keep and reach their optimal functional level in the physical, sensory, intellectual, psychological, and social aspects [8]. However, similar to other treatments, the effectiveness of orthotic treatment should be assessed using adequate evidence of its impact on different aspects of health [9]. At present, little information is available about the health status of Iranian veterans in the VMAF health care system. Much less is known about the use and usability of orthoses in veterans. Therefore, this study aimed to investigate the rate of using orthoses among Iranian veterans with neuromuscular and skeletal disorders of the ankle and foot. The rationale for choosing to look at ankle and foot disorders was their high prevalence among all war-related injuries [10]. However, the diversity of orthoses that are applicable for veterans with ankle and foot disorders may expand to all body parts because of the multiple war traumas that can occur in closed kinematic chains. Furthermore, this study explored the priorities of orthotic treatment in veterans with the ankle and foot disorders.

## Methods

**Fig. 1:**
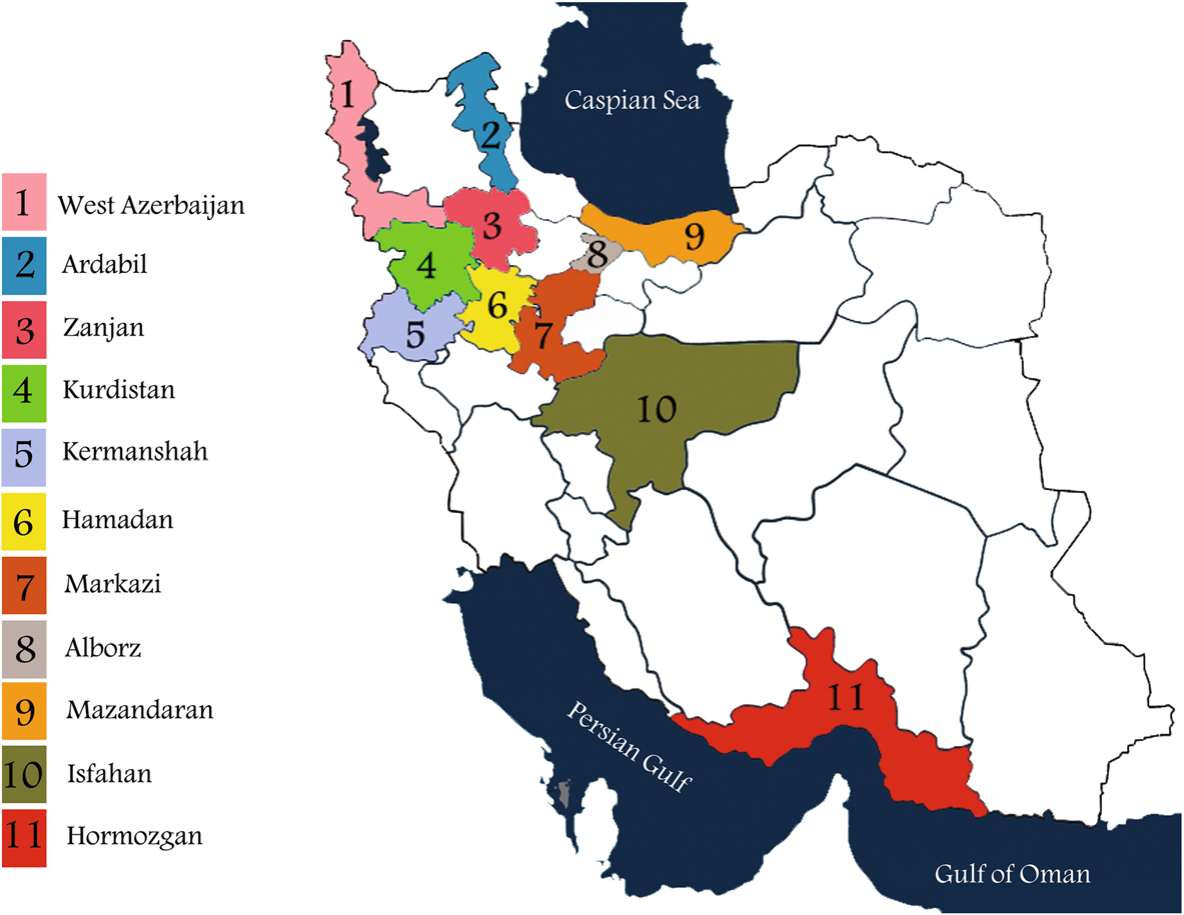
The eleven provinces of Iran in which the survey was conducted

This qualitative study was a national health needs assessment conducted by the Janbazan Medical and Engineering Research Center (JMERC) in 11 provinces of Iran from April 2011 to April 2016. Figure 1 shows those provinces in which the survey was conducted. The survey consisted of three distinct questionnaires: (a) demographic data, (b) a modified version of a physical self-maintenance scale, and (c) a self-developed orthotic needs assessment. The questionnaire for orthotic needs assessment was a comprehensive outcome measure that was developed using the Delphi method by a research team in JMERC to assess the current health status, rate of using orthoses, and orthotic needs of Iranian veterans. This questionnaire consisted of 30 close-ended and open-ended items that, in addition to health status, explored different aspects of orthosis use (i.e., the experience of orthosis use in daily activities, the quality of the current orthosis, reasons for withdrawal of orthosis, the use of assistive devices with an orthosis, and priorities for orthosis indication). The content validity of this questionnaire was approved by a multi-stakeholder research team during a pilot evaluation of 120 veterans before its application in the present study.

Because the care and self-care of veterans are multidisciplinary and lifelong issues, a stakeholder research group consisting of trained surveyors, an orthopedic surgeon, an occupational therapist, and an orthotist and prosthetist was established to complete the questionnaires [6]. The survey setup was arranged in such a way that distinct paper questionnaires for demographic data, the modified version of self-maintenance scale, and the orthotic needs assessment were filled in sequence by relevant experts of the research team for the participating veterans.

The participating veterans were selected using a judgmental sampling method from the VMAF database. The inclusion criteria were veterans with the existence of any neuromuscular or skeletal disorder of the ankle and foot and with the motivation to participate in the survey. Those selected veterans were invited to participate in 2–4 survey days in the capital city of each province. The number of days required to conduct the survey was determined based on the population of veterans who met the inclusion criteria in each province. On survey days, after trained surveyors gathered demographic data and took histories, the activities of daily living and gait quality were assessed by an occupational therapist. Thereafter, the health status of participant veterans was evaluated using clinical and orthopedic examinations by an orthopedic surgeon. Finally, the usability of orthoses and the quality of the current orthoses were evaluated by an orthotist and prosthetist. The exclusion criterion was the existence of cognitive disorder of the participant based on the evaluation by the occupational therapist. However, in the present study, there was no exclusion due to cognitive disorder. In addition, incomplete questionnaires with missing data were excluded. All aspects of the study were approved by the ethics committee of JMERC. Moreover, written informed consent was obtained from all veterans prior to enrollment.

Descriptive statistics were analyzed using SPSS, version 16.0. (SPSS Inc., Chicago, IL, USA) to report the frequency, percentage frequency, mean, and standard deviation of variables.

## Results

**Fig. 2:**
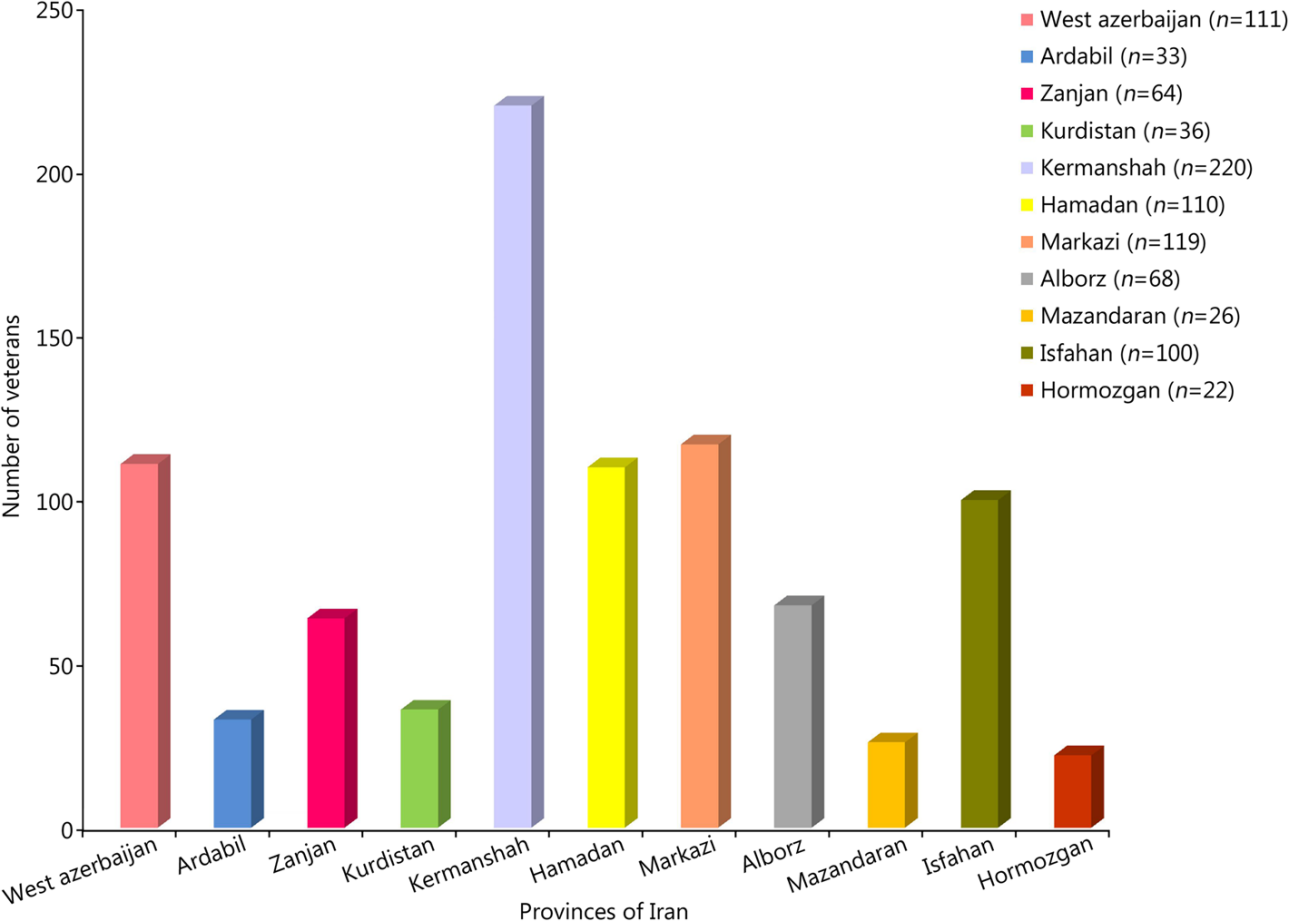
The frequency of participant veterans for the eleven provinces of Iran

Of the 2500 veterans with ankle and foot disorders who were invited to participate, 1124 enrolled in the study. In 217 cases, the questionnaires were incomplete and excluded from subsequent analyses. Therefore, the results of this study are reported based on the data obtained from 907 veterans (response rate: 80.7%). Figure 2 represents the frequency of participant veterans in the 11 provinces of Iran. As shown, the highest and lowest frequencies of participant veterans were in Kermanshah and Hormozgan provinces, respectively.

The demographic characteristics of participant veterans are presented in Table 1. Most of the veterans were men (*n* = 886; 97.68%), and their mean ± standard deviation (SD) of age and VMAF disability rate were 52.07 ± 8.13 and 31.92 ± 14.93, respectively.

**Table 1:** Demographic characteristics of veterans who participated in the survey

Variable	Frequency (Percentage)	Mean ± SD	Minimum-Maximum
Gender(*n* = 907)			
Male	886 (97.68)	–	–
Female	21 (2.32)	–	–
Age(year) (*n* = 907)	–	52.07 ± 8.13	7–93
Height(cm) (*n* = 907)	–	171.00 ± 7.70	90–193
Weight(kg) (n = 907)	–	79.13 ± 13.02	45–175
VMAF disability rating (%)(*n* = 907)	–	31.92 ± 14.93	5–70
Marital status(*n* = 907)			
Single	15 (1.65)	–	–
Married	892 (98.35)	–	–
Education level(*n* = 907)			
No education	73 (8.05)	–	–
Pre-diploma	424 (46.75)	–	–
Diploma	307 (33.85)	–	–
Bachelor of science	77 (8.49)	–	–
Master of science	18 (1.98)	–	–
Doctor of philosophy	8 (0.88)	–	–
Employment status(*n* = 907)			
Employed	378 (41.68)	–	–
Unemployed	529 (58.32)	–	–
Monthly compensation and pension received from VMAF^┼^(*n* = 907)			
Yes	423 (46.64)	–	–
No	484 (53.36)	–	–
Terrain condition around home (*n* = 898)			
Level	604 (67.26)	–	–
Uneven with ramp	196 (21.83)	–	–
Uneven without ramp	98 (10.91)	–	–
Climate condition in the living region(*n* = 907)			
Mild	480 (52.92)	–	–
Cold	346 (38.15)	–	–
Hot and dry	57 (6.28)	–	–
Hot and humid	24 (2.65)	–	–

-. No data; VMAF. Veterans and Martyrs Affair Foundation; ^┼^Compensation and pension from VMAF based on veteran’s disability rate

Almost all of the veterans (98.35%) were married, and most had pre-diploma and diploma educational levels. The unemployment rate in participant veterans was high (58.32%), and more than 46% of them were supported by the VMAF, receiving monthly compensation and pension based on their disability rating. Most of the veterans (67.26%) were living in regions with level terrain around their home. Moreover, the climate conditions were mild for more than 52% of the participating veterans.

The clinical characteristics of the participating veterans are presented in Table 2. Most of the participants (70.67%) had unilateral ankle and foot disorders. Additionally, nearly 50% of the veterans had associated disorders (e.g., chemical, neurological/mental, somatic, and combined form) in addition to their ankle and foot disorder. Neurological/mental disorder was the main associated disorder in participants.

**Table 2:** Clinical characteristics of veterans who participated in the survey

Variable	Frequency (Percentage)	Mean ± SD	Minimum-Maximum
Ankle/ft disorder(*n* = 907)			
Unilateral	641 (70.67)	–	–
Bilateral	266 (29.33)	–	–
Associated disorders in addition to ankle/ft disorder (*n* = 462)			
Chemical	50 (10.82)	–	–
Neurologic/mental	222 (48.05)	–	–
Somatic	99 (21.43)	–	–
Combination of all three types	91 (19.70)	–	–
Time since injury (year) (*n* = 907)			
0–10	11 (1.21)	–	–
11–20	20 (2.21)	–	–
21–30	410 (45.20)	–	–
31–40	466 (51.38)	–	–
Experience of orthosis use (*n* = 887)			
Has experience	379 (42.73)	–	–
Has no experience	508 (57.27)	–	–
How many days a week putting on orthoses (*n* = 208)	–	6.21 ± 1.68	0–7
Daily hours wearing orthosis (*n* = 208)	–	6.59 ± 3.75	0–20
Orthosis use during work (*n* = 378)			
Use	116 (30.69)	–	–
No use	262 (69.31)	–	–
Participation in social activities, sports, and recreation			
Nonparticipation	640 (70.56)	–	–
Participation	267 (29.44)	–	–
Using orthosis during social activities, sports, and recreation (*n* = 267)			
Use	93 (34.83)	–	–
No use	174 (65.17)	–	–
Fitting quality of the current shoe (*n* = 508)			
Unacceptable	291 (57.28)	–	–
Acceptable	217 (42.72)	–	–
Fitting quality of the current orthosis (*n* = 99)			
Unacceptable	54 (54.54)	–	–
Acceptable	45 (45.45)		
Scoliosis (*n* = 717)			
Yes	155 (21.62)	–	–
No	562 (78.38)	–	–
Kyphosis (*n* = 712)			
Yes	44 (6.18)	–	–
No	668 (93.82)	–	–
Hyperlordosis (*n* = 721)			
Yes	56 (7.77)	–	–
No	665 (92.23)	–	–
Iliac crest level (*n* = 705)			
Equality	431 (61.13)	–	–
Inequality	274 (38.87)	–	–
Leg length discrepancy (*n* = 907)			
Yes	639 (70.45)	–	–
No	268 (29.55)	–	–

-. No data

Of the 887 participant veterans who answered the question regarding their experience with orthosis use, 42.73% confirmed they had such an experience. The daily and weekly use of orthoses was 6.59 ± 3.75 h and 6.21 ± 1.68 days, respectively. Nearly 30% of the employed veterans used orthoses during work. The results revealed that the participation of veterans in social activities, sports, and recreation was weak (29.50%). Of those veterans who participated in social activities, sports and recreation, 34.83% were using a type of orthosis during such activities.

With respect to the fitting quality of the current shoe, the evaluation was conducted for 508 veterans. Nearly 57% of the veterans had unacceptable shoes based on the orthotist’s opinion. Similarly, the fitting quality of the current orthosis was unacceptable in nearly 54% of the veterans who were evaluated by an orthotist and prosthetist (*n* = 99). As seen from Table 2, leg length discrepancy (70.45%) and iliac crest inequality (38.87%) were the main clinical disorders in the participating veterans. The results of the physical self-maintenance scale (activities of daily living) are presented in Table 3. The type of the current orthosis in use, and the required orthosis and/or prosthesis are presented in Table 4.

**Table 3:** Results of the physical self-maintenance scale (*n* = 907)

Variable	Frequency (Percentage)
Feeding	
Does not feed self at all and resists efforts of others to feed him or her	5 (0.55)
Feeds self with assistance and is untidy	40 (4.41)
Eats without assistance	862 (95.04)
Bathing	
Needs assistance or supervision to wash and bathe face and hands and rest of body, respectively	157 (17.31)
Bathes self (tub, shower, sponge bath) without help	750 (82.69)
Grooming (neatness, hair, nails, hands, face, clothing)	
Needs assistance or supervision with grooming	158 (17.42)
Always neatly dressed and well-groomed without assistance	749 (82.58)
Dressing	
Completely unable to dress self and resists efforts of others to help	25 (2.76)
Needs assistance in dressing and selection of clothes	164 (18.08)
Dresses, undresses, and selects clothes from own wardrobe	718 (79.16)
Toileting	
No control of bowels or bladder	35 (3.86)
Needs to be reminded, needs help in cleaning self, or has soiling or wetting accidents while asleep or awake	116 (12.79)
Cares for self at toilet completely; no incontinence	756 (83.35)
Physical ambulation	
Sits unsupported in chair or wheelchair but cannot propel self without help	85 (9.37)
Ambulates with assistance of another person, railing, cane, walker, or wheelchair	168 (18.52)
Goes about grounds or city	654 (72.11)

**Table 4:** Type of current orthosis in use and the required orthosis and/or prosthesis for veterans who completed the orthotic need assessment questionnaire (n = 907)

Variable	Frequency (Percentage)
Types of participant’s current orthosis (*n* = 187)	
Medical shoe	99 (52.94)
Insole	42 (22.47)
Ankle foot orthosis	34 (18.18)
Knee orthosis	7 (3.74)
Knee ankle foot orthosis	3 (1.60)
Lumbosacral orthosis	2 (1.07)
Using walking assistive devices in addition to orthosis (*n* = 211)	
Yes	71 (33.65)
No	140 (66.35)
Type of assistive devices (*n* = 78)	
Cane	25 (32.05)
Elbow crutch	49 (62.82)
Arm crutch	4 (5.13)
Needs some type of lower limb orthoses on contralateral side (*n* = 865)	
Yes	320 (36.99)
No	545 (63.01)
Types of lower limb orthosis in need for contralateral side (*n* = 324)	
Medical shoes	26 (8.02)
Insoles	103 (31.79)
Ankle foot orthosis	18 (5.55)
Knee orthosis	172 (53.09)
Lumbosacral orthosis	2 (0.62)
Knee ankle foot orthosis	3 (0.93)
Lower limb prosthesis in need for contralateral side (*n* = 852)	
Yes	31 (3.64)
No	821 (96.36)
Lower limb prosthesis type in need for the contralateral side (*n* = 25)	
Syme prosthesis	6 (24.00)
Transtibial prosthesis	17 (68.00)
Transfemoral prosthesis	1 (4.00)
Knee disarticulation prosthesis	1 (4.00)
Needs some type of upper limb orthoses (*n* = 859)	
Yes	86 (10.01)
No	773 (89.99)
Type of upper limb orthoses in need (*n* = 109)	
Hand orthoses	12 (11.01)
Wrist hand orthoses	52 (47.71)
Elbow orthoses	21 (19.27)
Shoulder oorthoses	24 (22.02)
Needs some type of upper limb prostheses (*n* = 857)	
Yes	7 (0.82)
No	850 (99.18)
Type of upper limb prosthesis in need (n = 5)	
Finger and hand prosthesis	4 (80.00)
Transradial prosthesis	1 (20.00)
Needs some type of spinal orthoses (*n* = 860)	
Yes	273 (31.74)
No	587 (68.26)
Type of spinal orthosis in need (*n* = 275)	
Cervical collar	5 (1.82)
Sacral orthosis	2 (0.73)
Lumbosacral orthosis	261 (94.91)
Thoracolumbosacral orthosis	3 (1.09)
Bandage 8	4 (1.45)
Needs ocular and facial prostheses (*n* = 848)	
Yes	7 (0.83)
No	841 (99.17)
Needs some type of foot orthoses (n = 907)	
Yes	538 (59.32)
No	369 (40.68)
Needs some type of shoe (*n* = 907)	
Yes	447 (49.28)
No	460 (50.72)

Medical shoes and foot orthoses were the most used devices in the veterans. The results showed that 71 veterans were using walking assistive devices, mainly elbow crutches, in addition to their orthoses. Nearly 37% of the veterans needed some type of lower limb orthoses, mainly knee orthoses and insoles, on the contralateral side. Moreover, 31 veterans needed lower limb prostheses, mainly the transtibial type, for their contralateral side.

Nearly 10% of the participating veterans needed some type of upper limb orthoses. The wrist hand orthoses were the most required orthoses for participant veterans. There were 273 veterans in need of some type of spinal orthoses. Of those, 94.91% needed lumbosacral orthoses.

In sequential order, the most in-need orthoses for veterans were foot orthoses (*n* = 538), medical shoes (*n* = 447), lower limb orthoses on the contralateral side (*n* = 320), spinal orthoses (*n* = 273), and upper limb orthoses (*n* = 86).

## Discussion

The present study clearly showed the high demands for orthoses among Iranian veterans with ankle and foot disorders. The complexity of war injuries is due to their association with other disorders that may affect the whole life of a victim. Nearly 50% (*n* = 462) of the participating veterans were suffering from associated injuries in addition to their ankle and foot injuries. Considering the mean age of the participating veterans, 52.07 years, it can be expected that Iranian health care providers should focus more on providing facilities for elderly veterans in the near future. Uneven surfaces can make walking more difficult in people with disabilities and can potentially decrease their participation in social activities [11]. This problem can be resolved by preparing the urban environment for such people or providing them with high-tech lifts (e.g., in street crosswalks, the entrance of shopping centers, and gates of public transportation) [12]. Nearly 11% of the participating veterans were living in regions with uneven surfaces that did not have proper facilities for their ambulation. Although there are some supportive regulations for recruiting people with disabilities in public and private sectors [13], most of the participating veterans (~ 60%) were unemployed and preferred to adapt their living expenses to the monthly compensations and pensions that they received from VMAF. A possible reason for this might be the educational level of the participating veterans. The present survey revealed that most of the participants had pre-diploma and diploma educational levels and that less than 3% of them were willing to continue their education to the postgraduate level. Nearly 70% of those employed veterans were reluctant to use orthoses and assistive devices at work. This issue has its main roots in Iranian culture, as people with any type of disability prefer to mask it. Another reason might be the poor aesthetics of orthoses, which directly decreases the satisfaction of orthosis use [14]. The low participation (~ 30%) of veterans in social activities, sports, and recreation might indicate that they had few leisure activities. Therefore, the Iranian health care system should consider providing purposeful activities for veterans and those people with disabilities to promote their social participation.

The survey revealed that the most used orthosis in veterans was a medical shoe. However, based on the orthotist’s opinion, the quality of the shoe and its fit were dissatisfactory for nearly 57% of the veterans. Because footwear can directly affect balance during walking [15], the provision of proper shoes can prevent falls in veterans with ankle and foot disorders. Interestingly, the survey findings revealed that the most in-need orthoses were some type of foot orthoses (~ 59%) and shoes (~ 49%).

Ankle and foot disorders are common risk factors for falling and balance disturbance [16]. In addition, an asymmetry of the body can further increase the risk of falling. Therefore, due to the high prevalence of leg length discrepancy among veterans (*n* = 639), knee orthoses, insoles, and medical shoes are three main priorities of orthosis use for the contralateral side to compensate for ankle and foot disorders. In this regard, 71 veterans were using assistive devices other than orthoses to keep their balance and prevent falls. Furthermore, as found in the present survey, leg length discrepancy can lead to lower back pain, which can be controlled to some extent by spinal orthoses (~ 30%), i.e., lumbosacral orthoses (*n* = 261).

The results of the self-maintenance scale determined that physical ambulation was the most problematic activity for veterans. Fortunately, most of the veterans (> 72%) reported independence in feeding, bathing, grooming, dressing, toileting, and physical ambulation. The easiest activity for veterans was feeding (95.04%). Although the need for upper limb orthoses in our selected sample of veterans was low, the wrist hand orthoses were the most in-need orthoses (*n* = 52).

Considering that the availability of health services has an inverse relation with the health needs in a region [7], it simply may be inferred that the availability of orthoses is insufficient for Iranian veterans. Because there is a discrepancy between the current rate of orthosis use and the ideal situation, more resources should be provided for service providers to be able to serve veterans. As suggested by Klute et al. [6], the quality of health care can be improved by providing educational opportunities for veterans, enhancing communications with them, and developing remote monitoring systems in each province. Moreover, educating veterans regarding orthoses, their use, and their potential impacts on users’ life and health status is warranted.

There were some limitations associated with this survey. Limited resources were the main barriers to the ability to conduct this national survey in all provinces of Iran. In addition, the collaboration of some veterans was insufficient to prevent missing data and incomplete questionnaires.

## Conclusion

A national orthotic needs assessment was conducted via a systematic process to identify the health status (e.g., disability rating, demographics, associated injuries) and health care needs (e.g., activities of daily living, current orthosis in use, and priorities of orthosis use) in Iranian veterans with ankle and foot disorders. The findings of the survey revealed a discrepancy between the current health care status and the ideal one in respect to orthosis use in Iranian veterans. The findings of the present needs assessment can be used in strategic planning and decision making to improve the health care services provided to Iranian veterans.

## Abbreviations

JMERC: Janbazan Medical and Engineering Research Center
VMAF: Veterans and Martyrs Affair Foundation
